# Percutaneous Transforaminal Endoscopic Discectomy and Fenestration Discectomy to Treat Posterior Ring Apophyseal Fractures: A Retrospective Cohort Study

**DOI:** 10.1111/os.12698

**Published:** 2020-06-24

**Authors:** Yao‐bin Wang, Shu‐lian Chen, Chen Cao, Kai Zhang, Li‐min Liu, Yan‐zheng Gao

**Affiliations:** ^1^ Department of Orthopaedics Henan Provincial People's Hospital of Henan University Zhengzhou China

**Keywords:** Discectomy, Fenestration, Lumbar, Percutaneous transforaminal endoscopic, Posterior ring apophyseal fracture

## Abstract

**Objective:**

To compare the efficacy and safety of percutaneous transforaminal endoscopic discectomy (PTED) and fenestration discectomy (FD) for posterior ring apophyseal fractures (PRAF).

**Methods:**

This study was a retrospective cohort control study. A total of 96 patients with lumbar PRAF who underwent surgical treatment at the Henan Provincial People's Hospital of Henan University from September 2013 to December 2017 were retrospectively examined, of which 51 were treated by PTED and 45 by FD. The average age of those in the PTED group was 28.24 years, including 38 males and 13 females. The average age of those in the FD group was 28.07 years, with 33 males and 12 females. Operation time, total blood loss, hospitalization days, preoperative and postoperative visual analog scale (VAS), and Oswestry disability index (ODI) scores were evaluated. Modified MacNab criteria were used to evaluate the clinical effect at the last follow‐up.

**Results:**

Both operations were successful and no serious complications occurred. All patients were followed up for 12–30 (average 16.7 ± 3.2) months, and no patients were lost to follow‐up. No statistically significant difference was found in the mean age and gender between the PTED group and the FD group (*P* < 0.05). Operation time, total blood loss, and length of hospital stay were significantly lower in the PTED group (87.65 ± 13.15 min, 12.78 ± 4.95 mL, and 6.80 ± 1.67 days, respectively) than in the FD group (114.11 ± 14.39 min, 30.89 ± 7.09 mL, and 11.71 ± 1.98 days, respectively) (*P* < 0.05). The VAS and ODI scores of the two groups at postoperative day 1 (PTED: 3.82 ± 0.97, 37.73% ± 3.72%; FD: 3.62 ± 1.09, 36.62% ± 3.05%), and at 3 months (PTED: 2.90 ± 1.08, 26.02% ± 2.90%; FD: 3.07 ± 0.99, 27.16% ± 4.02%), 6 months (PTED: 2.31 ± 0.88, 22.53% ± 2.67%; FD: 2.36 ± 0.77, 21.18% ± 3.35%), and the last follow‐up (PTED: 1.90 ± 0.83, 19.88% ± 3.01%; FD: 1.89 ± 0.86, 18.22% ± 3.03%) were significantly different from the preoperative scores (PTED: 6.53 ± 1.00, 55.24% ± 4.54%; FD: 6.78 ± 1.31, 53.56% ± 5.73%) (*P* < 0.05). The VAS and ODI scores at 3 months postoperatively, 6 months postoperatively, and the last follow up were not significantly different between the two groups (*P* > 0.05). In the PTED group, 2 patients developed a transient nerve stimulation symptom within 1 day after surgery and 1 patient had recurrence at 3 months after surgery. In the FD group, 2 patients had severe dural ruptures due to adhesion during surgery, 1 patient developed infection complications, and 2 patients relapsed at 2 and 3 months after surgery. At the last follow‐up, the modified MacNab criteria for clinical effect were 93.3% and 94.1% in the FD and PTED groups, respectively.

**Conclusion:**

While PTED has the same efficacy as FD for treating PRAF, it is associated with shorter operation time, less trauma, and quicker recovery.

## Introduction

Posterior ring apophyseal fractures (PRAF) are uncommon. They are frequently accompanied by lumbar disc herniation and often seen in young adults^1^, ^2^. They cause corresponding symptoms of nerve root disorder or cauda equina nerve compression. Over the years, there have been many reports on PRAF^3^, ^4^, ^5^, ^6^, ^7^. Clinical manifestations of PRAF are similar to those of lumbar disc herniation, and there is no consensus on its pathogenesis. The most likely affected sites and disc levels of PRAF are consistent with lumbar disc herniation, which may be related to the close relationship between these two diseases 8. The reported incidence of lumbar posterior interruption is 5.7% in patients with lumbar disc herniation and is higher in children and adolescents, with rates up to 19% and 42%, respectively^4^. As most of these diseases affect young adults, the treatment principle should be the preservation of the normal structure and motor function of the spine.

The conservative treatment of PRAF is the same as for lumbar disc herniation, including bed rest, symptomatic treatment, and physiotherapy. Because the compression persists and the apophyseal fragment can cause spinal canal stenosis, the effect of conservative treatment is often poor. For patients with persistent or even aggravated symptoms, surgical treatment will be the best choice. As a treatment option, fenestration discectomy (FD) can be performed to resect the diseased intervertebral disc by removing the bone; however, it will inevitably cause damage to the normal spinal structure. While achieving satisfactory results, excessive destruction of the normal bony structure due to full exposure of calcified tissue will outweigh the gain. Different surgical methods determine the decompression approach, which, in turn, affects the postoperative spinal stability. Reducing surgical trauma, maintaining the stability of the spine after the operation, and avoiding the occurrence of adjacent spondylosis are problems that spinal surgeons need to pay attention to.

Since Kambin^9^ first reported the completion of arthroscopic microdiscectomy through “Kambin's triangle” in 1991, percutaneous endoscopic discectomy (PTED) has become one of the most popular minimally invasive spinal surgeries worldwide. PTED has been used in various types of lumbar disc herniation, lumbar spinal stenosis, and other diseases, and has demonstrated satisfactory clinical effects^10^, ^11^. PRAF is usually accompanied by prolapse of the lumbar intervertebral disc. PTED can puncture at the position of the osseous process and remove the herniated nucleus pulposus. PTED has the characteristics of less trauma, rapid recovery, and high safety in the treatment of lumbar disc herniation^10^, but there are relatively few reports on PTED in the treatment of PRAF. In the past 20 years, endoscopic treatment of PRAF was difficult. With the development of minimally invasive spinal instruments and the birth of endoscopic power systems such as grinding drills and ultrasonic bone knife, it is possible to remove bone fragments under endoscope. PTED is an effective surgical method for direct resection of intraspinal lesions, which can avoid destroying the normal bony structure and preserve motor function. This technique has gained increasing popularity as patient demand for more minimally invasive surgery has grown.

Although some scholars emphasize the importance of surgery, there is still no consistent surgical strategy for the surgical treatment of PRAF. Some scholars believe that when the herniated intervertebral disc is removed, the severed bone mass should be removed at the same time, to completely relieve the compression of the herniation at the nerve root or cauda equina. The residual bone mass can cause bony stenosis of the spinal canal and irreversible damage to nerve tissue^12^. However, some scholars believe that complete removal of the bone will prolong the operation time, destroy too many normal bone structures, and increase the difficulty of the operation, and can easily damage the nerve^13^. Only targeted direct decompression within the spinal canal can completely release the nerve root in cases with PRAF. Therefore, thorough removal of the bone mass that oppresses the nerve root and spinal canal is the key to achieving a satisfactory curative effect of FD in the treatment of PRAF. PTED treatment of PRAF should follow the principle of decompressing the nerve root as completely as possible without excessively destroying the normal bony structure to maintain the stability of the spine. However, due to the limited space under the endoscope, it is difficult to remove the severed bone mass completely, and serious neurological complications can even occur. Whether or not the apophyseal fragment is completely removed determines the scope of decompression, and affects the postoperative curative effect.

In this retrospective study, our objectives were to: (i) explore the feasibility of PTED in the treatment of lumbar PRAF; (ii) compare the efficacy and safety of PTED and FD in the treatment of lumbar PRAF; and (iii) explore whether it is necessary to remove the apophyseal fragment completely in PTED.

The study protocol adheres to the principles set forth by the 1964 Declaration of Helsinki and its later amendments.

## Materials and Methods

### *Inclusion and Exclusion Criteria*

Inclusion criteria: (i) PRAF patients hospitalized in the orthopaedics department of our institution from September 2013 to December 2017; (ii) patients who underwent PTED or FD treatment; (iii) visual analog scale (VAS), Oswestry disability index (ODI), and the MacNab score were compared; (iv) the related outcomes of patients were completely recorded; and (v) a retrospective cohort study.

Exclusion criteria: (i) patients whose imaging findings were not consistent with the symptoms and signs of PRAF; (ii) patients who have serious neurological deficit and/or spinal instability; (iii) patients with lumbar spondylolisthesis of grade II or higher; (iv) patients with elevated infection indicators, including erythrocyte sedimentation rate and C‐reactive protein; and (v) patients with lumbar trauma, cancer or other serious systemic diseases, or who were lost to follow up.

### *Group Allocations*

The PTED group had 51 patients (38 men and 13 women) and the FD group had 45 patients (33 men and 12 women). Both surgeries were performed by the same spine surgeon.

### *Surgical Procedure*

#### *Percutaneous Transforaminal Endoscopic Discectomy Group*

##### Anesthesia and Position

Surgeries were performed under local anesthesia with patients in the prone position on a radiolucent operating table. Patients lay on the unaffected side with their legs flexed.

##### Approach and Exposure

The entrance point was located superior to the iliac crest, approximately 10–14 cm from the midline. After the local infiltration of lidocaine, an 18‐gauge needle was introduced from the entrance point to the lateral foramen under C‐arm fluoroscopy guidance. A 22‐gauge needle was then inserted through the 18‐gauge needle into the herniated disc, followed by the injection of a contrast medium (9 mL of iohexol with 1 mL of methylene blue) into the disc. Thereafter, the 22‐gauge needle was removed, and a guide wire was inserted *via* the 18‐gauge needle. Subsequently, an 8‐mm incision was made in the area of the guide wire, dilators were inserted consecutively, and reamers were used to dilate the bony foramen appropriately.

##### Decompression

The working cannula, through which the endoscope with the working channel and irrigation systems was inserted, was advanced along the dilator. The blue‐stained degenerated disc material was then identified and removed using endoscopic forceps until sufficient decompression of the nerve root was achieved.

##### Close

The working cannula and endoscope were removed following adequate hemostasis, and the skin was finally sutured.

#### *Fenestration Discectomy Group*

##### Anesthesia and Position

Surgeries were performed under general anesthesia with patients in the prone position on a radiolucent operating table.

##### Approach and Exposure

A midline skin incision was made, and paravertebral muscles were divided. The lower edge of the upper vertebral body and medial edge of the inferior articular process were excised, and the ligamentum flavum was pushed using a bone curette to dissect the ligamentum flavum. Peeling and resection were performed carefully to avoid tearing and contusion of the dural sac and nerve root. Some laminae were clamped by the laminectomy forceps.

##### Decompression

The dural sac and nerve root were pulled to one side using the nerve retractor, and excessive nerve traction was avoided. The prominent nucleus pulposus and posterior edge of the vertebral body were fully exposed. After protecting the nerve root and stopping bleeding adequately, the posterior bone margin was removed and chiseled if necessary. Subsequently, the area was checked for adequate decompression of the lateral recess and the nerve root canal.

##### Close

The incision was washed, and it was confirmed that there was no active bleeding. The drainage tube was placed, and the incision was sutured.

### *Outcome Measures*

Operative reports and medical charts were reviewed to obtain preoperative and postoperative clinical data, including operation time, total blood loss, and hospitalization days. Visual analog scale (VAS) and Oswestry disability index (ODI) scores^14^ were recorded before operation, on postoperative day 1, at 3 months, at 6 months, and at the last follow up. At the last follow up, the MacNab score^15^ was used to evaluate surgical efficacy.

#### *Visual Analog Scale*

The VAS is the most commonly used questionnaire for quantification of pain. It is a continuous scale comprised of a horizontal or vertical line, usually 10 cm in length. For pain intensity, the scale is most commonly anchored by “no pain” (score of 0) and “pain as bad as it could be” (score of 10). A score of 0 is considered no pain, 1–3 mild pain, 4–6 moderate pain, and 7–10 severe pain.

#### *Oswestry Disability Index*

The Oswestry disability index (ODI) is one of the principal condition‐specific outcome measures used in the management of spinal disorders, and to assess patient progress in routine clinical practice. The ODI score system includes 10 sections: pain intensity, personal care, lifting, walking, sitting, standing, sleeping, sex life, social life, and traveling. For each section of six statements, the total score is 5. Intervening statements are scored according to rank. If more than one box is marked in each section, the highest score is taken. If all 10 sections are completed, the score is calculated as follows: total scored out of total possible score × 100. If one section is missed (or not applicable), the score is calculated as: total score/(5 × number of questions answered) × 100%. A score of 0%–20% is considered mild dysfunction, 21%–40% is moderate dysfunction, 41%–60% is severe dysfunction, and 61%–80% is considered a disability. For cases with a score of 81%–100%, patients are either long‐term bedridden, or exaggerating the impact of pain on their life.

#### *Modified MacNab Criteria*

The modified MacNab criteria were used to evaluate the efficacy of surgery. The modified MacNab criteria include four grades: excellent, good, fair, and poor. Excellent: symptoms disappear completely, return to the original work and life; good: mild symptoms, activity is slightly limited, no impact on work and life; fair: symptoms are relieved, activities are limited, affecting normal work and life; poor: there is no difference before and after treatment, even aggravated.

## Statistical Analysis

Statistical analysis was performed using SPSS 22.0 software (IBM, Armonk, NY, USA). Statistical methods were used to compare patient demographic data and clinical outcomes of the two groups. Continuous and categorical parameters were analyzed using the *t*‐test and the χ^2^‐test, respectively. A *P*‐value <0.05 was considered statistically significant.

## Results

### *Follow Up*

Surgeries were successfully completed in both groups. All patients were followed up for 12–30 months, with an average follow up of 16.7 ± 3.2 months.

### *General Results*

No statistically significant differences were found in the mean age of patients in the two groups at the time of surgery (PTED group at 28.24 years *vs* FD group at 28.07 years, *P* = 0.376) and the sex ratio (male : female ratio was 38:13 in the PTED group *vs* 33:12 in the FD group, *P* = 0.896). Compared with the FD group, the average blood loss, the average operation time, and the average hospital stay in the PTED group were significantly lower than those in the PTED group (all *P* < 0.0001) (Table 1).

**Figure 1:**
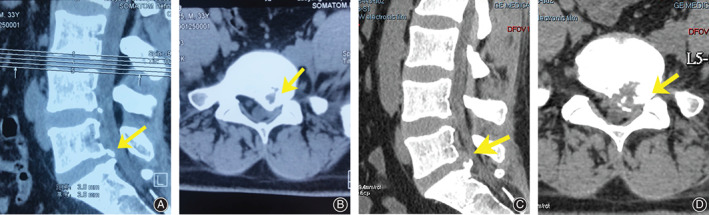
A 33‐year‐old male patient was hospitalized for pain and numbness of the left lower extremity for 6 months. (A & B) Preoperative CT images; his diagnosis was L_5_S_1_ posterior ring apophyseal fracture. (C & D) Postoperative CT images. After percutaneous transforaminal endoscopic discectomy, most of the bones were removed, and a small amount of bone remained. Soft tissue was removed and symptoms of compression were relieved.

**Figure 2:**
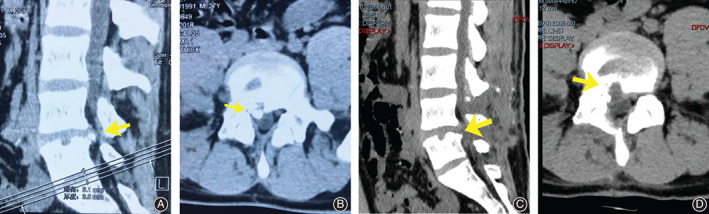
A 27‐year‐old male patient was hospitalized for low back pain and right lower extremity pain for 4 months. (A & B) Preoperative CT images; his diagnosis was L_4–5_ posterior ring apophyseal fracture. (C & D) Postoperative CT images. After FD, the bone was removed, and the compression was released.

For the patients in group PTED, the posterior stable bone mass remained a little, and the symptoms were relieved (Fig. 1). For the patients in group FD, decompression was complete and symptoms were relieved (Fig. 2).

**Table 1:** Summary of clinical outcomes of patients who underwent PTED and FD (Mean ± SD)

Surgery	Operating time (min)	Total blood loss (mL)	Admission days (d)
PTED	87.65 ± 13.15	12.78 ± 4.95	6.80 ± 1.67
FD	114.11 ± 14.39	30.89 ± 7.09	11.71 ± 1.98
*P‐*value	0.000	0.000	0.000

FD, fenestration discectomy; PTED, percutaneous transforaminal endoscopic discectomy.

### *Visual Analog Scale*

In the PTED group, the VAS scores after 1 day (3.82 ± 0.97), and at 3 months (2.90 ± 1.08), 6 months (2.31 ± 0.88), and the last follow up (1.90 ± 0.83) were significantly lower than those before surgery (6.53 ± 1.00) (*P* < 0.05). In the FD group, the VAS scores at 1 day (3.62 ± 1.09), and at 3 months (3.07 ± 0.99), 6 months (2.36 ± 0.77), and the last follow up (1.89 ± 0.86) were significantly lower than those before surgery (6.78 ± 1.31) (*P* < 0.05).

The preoperative VAS scores of the two groups were 6.53 ± 1.00 and 6.78 ± 1.31, respectively, and there was no significant difference between the two groups (*P* > 0.05). There was no significant difference in VAS scores between PTED and FD groups at 1 day (3.82 ± 0.97 *vs* 3.62 ± 1.09), and at 3 months (2.90 ± 1.08 *vs* 3.07 ± 0.99), 6 months (2.31 ± 0.88 *vs* 2.36 ± 0.77) and the last follow up (1.90 ± 0.83 *vs* 1.89 ± 0.86) (*P* > 0.05) (Table 2).

**Table 2:** Comparison of preoperative and postoperative visual analog scale scores (VAS) between the two groups (Mean ± SD)

Surgery	Preoperation	Postoperative 1 day	Postoperative 3 months	Postoperative 6 months	Last follow up
PTED	6.53 ± 1.00	3.82 ± 0.97*	2.90 ± 1.08*	2.31 ± 0.88*	1.90 ± 0.83*
FD	6.78 ± 1.31	3.62 ± 1.09*	3.07 ± 0.99*	2.36 ± 0.77*	1.89 ± 0.86*
*P*‐value	0.085	0.194	0.433	0.370	0.541

Note: Compared with preoperative scores.

* *P* < 0.05.

FD, fenestration discectomy; PTED, percutaneous transforaminal endoscopic discectomy

### *Oswestry Disability Index*

In the PTED group, the ODI scores after 1 day (37.73% ± 3.72%), and at 3 months (26.02% ± 2.90%), 6 months (22.53% ± 2.67%), and the last follow up (19.88% ± 3.01%) were significantly lower than those before surgery (55.24% ± 4.54%) (*P* < 0.05). In the FD group, the ODI scores after 1 day (36.62% ± 3.05%), and at 3 months (27.16% ± 4.02%), 6 months (21.18% ± 3.35%), and the last follow‐up (18.22% ± 3.03%) were significantly lower than those before surgery (53.56% ± 5.73%) (*P* < 0.05).

The preoperative ODI scores of the two groups were 55.24% ± 4.54% and 53.56% ± 5.73%, respectively, and there was no significant difference between the two groups (*P* > 0.05). There was no significant difference in ODI scores between PTED and FD groups after 1 day (37.73% ± 3.72% *vs* 36.62% ± 3.05%), and at 3 months (26.02% ± 2.90% *vs* 27.16% ± 4.02%), 6 months (22.53% ± 2.67% *vs* 21.18% ± 3.35%), and the last follow‐up (19.88% ± 3.01% *vs* 18.22% ± 3.03%) (*P* > 0.05) (Table 3).

**Figure 3:**
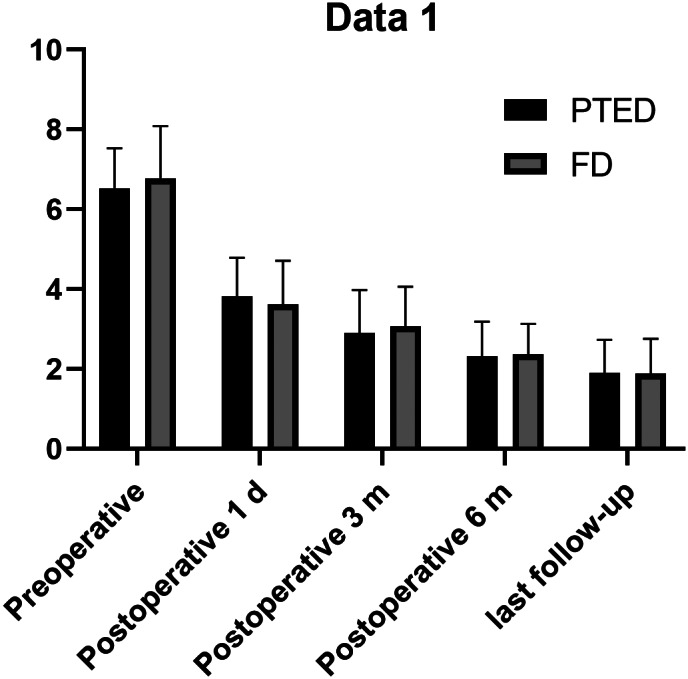
The course of the pain in both groups, which was rated using the mean visual analog scale. FD, fenestration discectomy; PTED, percutaneous transforaminal endoscopic discectomy.

**Table 3:** Comparison of preoperative and postoperative Oswestry disability index (ODI) scores between the two groups (Mean ± SD, %)

Surgery	Preoperation	Postoperative 1 day	Postoperative 3 months	Postoperative 6 months	Last follow up
PTED	55.24 ± 4.54	37.73 ± 3.72*	26.02 ± 2.90*	22.53 ± 2.67*	19.88 ± 3.01*
FD	53.56 ± 5.73	36.62 ± 3.05*	27.16 ± 4.02*	21.18 ± 3.35*	18.22 ± 3.03*
*P‐*value	0.096	0.112	0.082	0.240	0.920

Note: Compared with preoperative.

* *P* < 0.05.

FD, fenestration discectomy; PTED, percutaneous transforaminal endoscopic discectomy.

**Figure 4:**
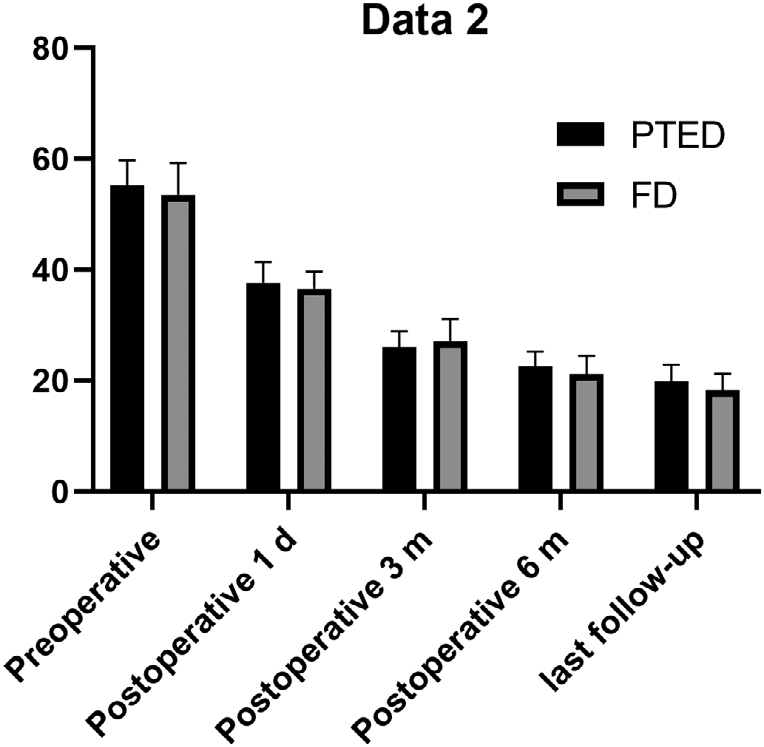
The course of the lumbar function in both groups, which was rated using the Oswestry disability index. FD, fenestration discectomy; PTED, percutaneous transforaminal endoscopic discectomy.

### *Modified MacNab Criteria*

At the last follow up, the modified MacNab criteria were used to evaluate the curative effect. The satisfaction rate for the modified MacNab criteria in the FD group was 93.3%. The satisfaction rate for the modified MacNab criteria in PRAF group was 94.1%.

### *Complications*

In the PTED group, 2 patients developed a transient nerve stimulation symptom within 1 day after surgery and recovered well after active treatment. One patient had recurrence after surgery. This patient had a long course of PRAF with severe lumbar disc calcification. The PRAF was located in the center, and removing the bone was not easy. After conservative treatment for 1 month without significant effect, the patient underwent FD and recovered well after surgery.

In the FD group, 2 patients had severe dural rupture due to adhesion during surgery, 1 patient developed infection complications, and 2 patients relapsed 2 and 3 months after surgery. Following active treatment, they recovered well.

## Discussion

Fenestration discectomy, a common surgical technique for PRAF, is known to relieve nerve root compression. In contrast, as direct puncture can be done during PTED to reach the anatomical location of the PRAF, the structure of the spine can be preserved to the maximum extent. The primary purpose of this study was to compare the efficacy and safety of these two surgical methods. In this study, we observed no significant difference in the VAS and ODI scores between the FD and PTED groups after surgery. However, there was a significant difference in the mean blood loss volume, mean operation time, and mean hospital stay between the two groups.

### *Pathological and Imaging Features of Posterior Ring Apophyseal Fractures*

In 1973, Lowrey^16^ first reported three cases of adolescent lumbar vertebral epiphysis protruding into the vertebral canal that compressed the nerve roots; since then, many scholars^1^, ^2^, ^17^ have successively studied the pathogenesis of this disease. Some scholars^18^ believe that during the development in the adolescence period, some anatomical defects occur in the back of the vertebral body due to delayed or defective development of the vertebral body, which forms a stress concentration area. After a traumatic or repeated chronic injury, some anatomical defects in the posterior cartilage plate of the vertebral body lead to rupture of the cartilage plate and formation of fissures, which are caused by stress concentration in the posterior cartilage plate of the vertebral body.

The nucleus pulposus slips into the intervertebral body and epiphysis through a fissure, forming Schmorl nodules, which squeezes and subsequently displaces the epiphysis backward. With increasing age, growth of the spine halts, epiphysis ossifies, and intervertebral disc degenerates gradually. The weakened elasticity of the annulus fibrosus cannot further hold the moving nucleus pulposus, thereby the bone mass moves backward or the bone mass and vertebral body are completely separated or only connected. This condition causes spinal canal stenosis or lateral recess stenosis, compressing the cauda equina and the nerve root, which manifests as a series of clinical symptoms similar to those of lumbar disc herniation or spinal canal stenosis.

Routine lumbar X‐ray imaging cannot easily detect PRAF; however, CT can clearly show the shape, the location of the lumbar disc herniation and PRAF, and the extent of space occupied by the lumbar canal. CT is the most effective imaging modality for diagnosing PRAF^19^, ^20^. MRI can clearly show the location, size, and degree of nerve compression of the herniated intervertebral disc. The bone mass protruding into the spinal canal at the posterior edge of the vertebral body, protruding calcified discs, and ossification of the posterior longitudinal ligament and osteophyte at the posterior edge of the vertebral body should be identified. The latter is not accompanied by a bone defect at the posterior edge of the vertebral body. In addition, the bone defect of the posterior edge of the vertebral body should be distinguished from bone destruction lesions such as tuberculosis and tumors of the vertebral body.

### *Feasibility of Percutaneous Transforaminal Endoscopic Discectomy for Posterior Ring Apophyseal Fractures*

Posterior ring apophyseal fractures often need surgical treatment. Chang *et al*. ^1^ report 12 cases of PRAF treated conservatively. Half of the patients had to undergo surgical decompression because of epiphyseal ring rupture and compression symptoms. Surgical treatment is the most effective and main treatment for PRAF^21^. Currently, the choice of surgical method for lumbar PRAF is controversial. Although traditional open surgery can achieve a satisfactory neuro‐decompression effect, the operation is traumatic and will inevitably destroy the normal structure and motor function of the lumbar spine, which is likely to lead to long‐term accelerated degeneration of the adjacent segments and vertebral diseases^22^. With the development in percutaneous spinal endoscopy technology, indications for surgery have been gradually expanded^8^, ^23^. Percutaneous spinal endoscopy has been used in the treatment of various types of lumbar disc herniation and spinal stenosis^24^, ^25^. Surgery involving the intervertebral foramen is often associated with short operation time, less bleeding, and fewer complications, as this structure is closer to the anatomical position of the PRAF bone block; that is, the anterior epidural space. Jasper *et al*.^26^ argue that percutaneous foramen endoscopy causes minimal iatrogenic damage to the nerve roots because of its inherent characteristics of targeting the puncture concepts and expanding the foramen.

With the improvement in spinal minimally invasive techniques and the accumulation of surgical experience, we believe that percutaneous transforaminal endoscopic discectomy is also suitable for the treatment of PRAF. Endoscopic ring saws and electric drill systems help to remove calcified lesions. The authors in that study used PTED to treat various types of lumbar disc herniation in the early stage and found that discectomy under endoscopy can achieve effective decompression and remove bone protrusions. In young and middle‐aged patients with PRAF, surgery should maintain the normal structure of the lumbar spine as much as possible while ensuring adequate decompression and avoiding iatrogenic lumbar instability.

### *Efficacy of Percutaneous Transforaminal Endoscopic Discectomy for Posterior Ring Apophyseal Fractures*

The VAS and ODI scores of the two groups on postoperative day 1, and at 3 months, 6 months, and the last follow up were significantly different from the preoperative scores (*P* < 0.05) (Figs. 3, 4). PTED has the advantages of less trauma and a reliable decompression effect. In this study group, under clear vision, the posterior border of the vertebral body could be resected successfully, and the nerve root could be safely protected. The authors believe that PTED has the following advantages: first, tissue damage is minimal and does not require extensive exfoliation of the paravertebral muscles; second, the amount of intraoperative bleeding is small, at 3–10 mL; and, third, the normal structure of the spine is less damaged, and the middle and rear column structures are completely preserved, which does not affect the stability of the spine. Compared with FD, the operation time is significantly shortened and the recovery time is shorter. Patients can wear an abdominal binder and get out of bed on the day after surgery. They can be discharged from hospital 2–3 days after surgery, and resume normal work and life after 1 month. PTED reduces patient physical burden and hospitalization costs^14^.

### *Strategies of Percutaneous Transforaminal Endoscopic Discectomy*

Percutaneous transforaminal endoscopic discectomy treatment of PRAF is relatively difficult, and the learning curve is steep^9^. For beginners, experience in open surgery experience is required. Puncture positioning requires that the surgeon understands anatomical structures and can convert two‐dimensional images of the brain into three‐dimensional images. In addition, according to the position of the ankle ring, during the formation of the superior articular process, surgeons should retain the articular surface, in addition to sawing the bone of the ventral side of the upper joint, if necessary. The upper edge of the lower vertebral body can be sawed. Moreover, to adjust the position of the working cannula, the vertebral part of the bone can be manipulated horizontally to remove the free disconnected ankle ring.

When the decompression is completed, the nerve root should be synchronized with the heart. In addition, the straight leg raising test allows not only the surgeon to observe the nerve root activity but also the patient to compare preoperative and postoperative functions. In our experience, we inserted a radiofrequency ablation electrode into the intervertebral disc for multi‐point ablation coagulation, which not only eliminated painful stimuli but also created favorable conditions for the healing of the annulus fibrosus. The radiofrequency electrode eliminates nerve ending receptors that grow within the broken annulus. At the same time, intraoperative continuous perfusion with saline flushes out toxic metabolites in the intervertebral disc to prevent the accumulation of electrocoagulation by‐products during surgery^12^.

Whether there is a need for complete resection of the bone behind PRAF is currently controversial. Some scholars believe that the operation should completely remove the bone and intervertebral disc tissue at the posterior edge of the vertebral body and completely relieve the compression of the nerve root or cauda equina. However, some scholars believe that the stable bone behind the PRAF vertebral body, if not causing spinal stenosis and nerve root compression, does not need to be completely removed to maintain more posterior column composite structure and unruptured intervertebral disc tissue and to maintain the stability of the spine as much as possible. The author believes that free bones should be removed as much as possible. If nerve root compression and spinal stenosis are not caused by the stable bone mass, complete removal of the bone mass is not required.

### *Limitations*

First, the inherent limitation of the study's retrospective design should be considered. Second, its long‐term efficacy and complications need to be further observed and studied. Based on the results of this study, it is our goal to conduct randomized control studies in the future.

### *Conclusion*

In conclusion, PTED has the same efficacy as FD in the treatment of PRAF but has the advantages of shorter operation time, less trauma, and quick recovery. PTED reduces trauma to the spine structure while treating PRAF, which is especially important for young and middle‐aged patients. However, its long‐term efficacy and complications need to be further observed and studied.
